# N-Glycosylation Is Required for Secretion and Mitosis in *C. elegans*

**DOI:** 10.1371/journal.pone.0063687

**Published:** 2013-05-14

**Authors:** Julia Stevens, Anne Spang

**Affiliations:** Growth & Development, Biozentrum, University of Basel, Basel, Switzerland; University of North Carolina at Chapel Hill, United States of America

## Abstract

N-glycosylation of proteins is an essential process, and N-glucans serve as important beacons in protein folding and ER associated degradation. More importantly, N-glycosylation increases the structural repertoire of proteins because the addition of the N-glucan on proteins will serve as a base for further sugar additions in the Golgi apparatus, and hence complex three-dimensional structures can be build. N-glycosylation is mediated by the ER-resident OST complex, which is essential throughout eukaryotes. Partial knockdown of conserved OST complex members, such as *C. elegans* RIBO-1, led to an embryonic lethal phenotype. Although the ER morphology was not grossly altered in *ribo-1(RNAi)* oocytes and embryos, secretion of yolk and of the yolk receptor RME-2 was perturbed in those worms. Perhaps as a consequence of reduced arrival of N-glycosylated proteins at the plasma membrane, cytokinesis occurred less efficiently leading to multinuclear cells. Unexpectedly, we detected a chromosome segregation defect in *ribo-1(RNAi)* embryos suggesting an essential role of at least one N-glycosylated protein in metaphase-anaphase transition.

## Introduction

N-glycosylation is the most abundant post-translational modification of proteins that enter the secretory pathway at the endoplasmic reticulum (ER). Proteins of the secretory pathway are synthesized on ribosomes associated with the ER-resident Sec61 translocon, through which the nascent polypeptide chain passes in an unfolded state. In the ER lumen an N-glucan, consisting of 2 N-acetylglucosamine, 9 mannose, and 3 glucose residues, is transferred onto an asparagine residue of the polypeptide chain, which fits the sequence signature N-X-S/T (X can be any residue except proline). The sugars are covalently linked to the asparagine through an amide bond by the evolutionary conserved oligosaccharyl transferase (OST) complex [1].

The folding process of a nascent polypeptide chain starts with its arrival in the ER lumen and is usually assisted by chaperones; the added N-glucan will impact the 3D structure of the folded protein. In addition, the state of the N-glucan helps the cell to determine whether a protein is correctly folded and can move on to the Golgi apparatus or whether a protein should be retrotranslocated into the cytoplasm and be degraded by the proteasome [2]. In case the polypeptide chain has adopted a correct fold, it may exit the ER through COPII-coated vesicles and reach the Golgi apparatus, in which the N-glycan is modified extensively and rather complex sugar trees can be build. These sugar trees are important for proper protein function and provide in addition specific protein-protein interaction sites.

In yeast, nine components of the OST complex have been identified (Table 1). However, they are probably organized in at least two distinct complexes [3]–[5]. The human OST complex appears to comprise seven subunits, all of which are conserved in yeast; but here again more than one OST complex exist, which may differ in client specificity. Five members of the yeast OST complex (Ost1p, Swp1p, Stt3p, Ost2p and Wbp1p) are essential for viability and can be considered as core of the OST complex. Ribophorin I (RPNI)/Ost1p recognizes the N-glycosylation signal on the nascent polypeptide chain, while STT3 represents the catalytic subunit of the complex. OST48 appears to link RPN2 and DAD1 to RPNI [6].

**Table 1:** Homologs of OST complex members in different species.

*C. elegans*	*S. cerevisiae*	*H. sapiens*
*ribo-1*	*OST1*	RPN I
*ostd-1*	*SWP1*	RPN II
*stt-3*	*STT3*	STT3-A/STT3-B
*dad-1*	*OST2*	DAD1
*ostb-1*	*WBP1*	OST48
ZK686.3	*OST3*	N33/TUSC3, IAP, DC2
?	*OST4*	OST4
?	*OST5*	?
?	*OST6*	DC2
?	*?*	KCP2

Despite the knowledge of the OST complex in yeast and mammals, very little is known about the role of N-glycosylation in the development of a multicellular organism. Here, we show that the OST complex is essential for secretion and endocytosis because of impaired trafficking of the yolk receptor RME-2 in oocytes and of yolk secretion defects from the gut. In addition the fertilized embryos were osmo-sensitive probably due to a reduction of the secretion of egg-shell material. Finally, reducing N-glycosylation of proteins caused a defect in cytokinesis. Most surprisingly however, we found that knockdown of *ribo-1* caused failure to properly attach and/or segregate chromosomes during mitosis, thus describing a novel role for N-glycosylation in mitosis. This report provides an unexpected link between N-glycosylation activity and chromosome segregation and thus to cancer development.

## Materials and Methods

### General Methods and Strains

*C. elegans* was cultured and maintained as described previously [7] at 20°C. The Bristol N2 strain was used for embryonic lethality experiments, embryo lysate and WGA-Qdot-staining. Strains AZ212 (unc-119(ed3) ruIs32 III [*pie-1::GFP::H2B+unc-119(+)*]) [8], WH204 (unc-119(ed3) III; ojIs1[*pie-1::GFP::tbb-2+ unc-119(+)*]) [9] and DH1033 (sqt-1(sc103) II; bIs1 [*vit-2::GFP+rol-6(su1006)*] X) [10] were obtained from the *Caenorhabditis* Genetics Center (CGC). Strain WH327 (unc-119(ed3) III; ojIs23 [*pie-1::GFP::SP12 unc119(+)*]) [11] was created by Jayne Squirrell. Barth Grant provided strain RT408 (unc-119(ed3) III; pwIs116 [*rme-2::GFP+unc-119(+)*] [12]), and strain XA3507 (unc-119(ed3) qaIs3507 III [*pie-1::GFP::lem-2+ unc-119(+)*]) [13] was contributed by Ian Mattaj.

### RNAi Experiments

For construction of the feeding vectors, the sequences of the OST genes were obtained by PCR from cosmids, genomic or cDNA with the following primers:

ribo-1 fwd 5′-gcgattgctatttgccatcgctccctggg-3′.

ribo-1 rev 5′-ACATGTTCTGGAAGAAGAACTTTAGTG-3′.

ostd-1 fwd 5′-AAGCTACTTCTTGTGCTCCTGA-3′.

ostd-1 rev 5′-CTACTCTGATTTTTTTGCTTTCG-3′.

dad-1 fwd 5′-GGCGGCTCAAGTATTCCAGTTCTCTCG,

dad-1 rev 5′-TCCCAAGAAGTTGACGACGACAA-3′.

stt-3 fwd 5′-GACATCAACAACGGCGGCTCGAAC-3′.

stt-3 rev 5′-AGCTTTAGAAGCGGTTGGAGCTGGTCG-3′.

The PCR products were sub-cloned using the TOPO® TA Cloning® Kit according to manufacturer’s protocol (Life Technologies), cloned into plasmid L4440 and transformed into *e. coli* HT115 [14]. RNAi was performed as described [15]. L4-staged larvae were cultured on the plates for 24–36 hours at 23°C prior to analysis of their oocytes and embryos. For RNAi by injection, the L4440-constructs were used to PCR-amplify the insert together with the adjacent T7-sites using a standard T7-primer. The gel-purified PCR products were used as templates for *in vitro* transcription using T7 polymerase (Promega). dsRNA was produced according to the manufacturer’s protocol (Promega), purified by phenol/chloroform extraction and resuspended in 20 µl RNase-free water. dsRNA was injected into the gonad or body cavity of young adult worms, which were subsequently incubated at 20°C. The progeny of the injected animals was analyzed. For the determination of embryonic lethality, 5 to 15 L4 larvae or injected young adults were singled out on RNAi plates or NGM plates containing *e. coli* strain OP50 as food source, and transferred every 24 hours to fresh plates until they stopped egg laying, usually 2–4 days at 20°C. For each plate, the amount of larvae and non-hatched eggs was determined 24 hours after removal of the adult.

### Lysate Preparation and Immunoblot Analysis

For preparation of embryo lysates, synchronized L4 larvae were plated on 15 mock or RNAi plates (6 cm) and grown for 24 to 36 hours at 23°C. The next day, adult worms were washed off the plates with egg buffer, allowed to settle by gravity flow and washed 3 times with egg buffer. Since the protein content of the permeable RNAied embryos would have been destroyed by bleaching, the concentrated worms were transferred to a 1 ml tissue grinder (Wheaton) and dounced with a tight pistil for about 10 to 15 strokes. Unbroken worms and carcasses were allowed to settle for 1 min, and the supernatant containing the released embryos was collected. After repeating this for 5 to 10 times, the combined supernatants were filtered through a 10-µm nylon net filter (Millipore) inserted into a 40 µm cell strainer (BD) placed on top of a Steriflip 50 ml filtration unit (Millipore) attached to a vacuum pump. This way we removed small debris from the carcasses and bacteria that were washed out of the intestines. The embryos were rinsed with 10 ml egg buffer before being washed off the nylon net in 1 ml egg buffer and pelleted by centrifugation. The proteins were extracted using Trizol (Life Technologies) according to the manufacturer’s protocol. Standard methods were applied for SDS-PAGE and Coomassie staining, and ProQ®Emerald 300 staining was performed according to the manufacturer’s protocol. For gonad lysate, 5 RNAi- or mock-treated adults were cut behind the pharynx in a deep-well slide containing 10 µl egg buffer completed with 0.01% TX100, 1 mM PMSF, 1× complete, EDTA-free protease inhibitor cocktail (Roche), 1 mM EDTA and 2 mM levamisole. The extruded gonad arms were cut off at the spermatheca, the carcasses quickly removed using an eyelash, and the isolated gonads were then mouth-pipetted into tubes on ice. Five µl Laemmli-buffer were added to the gonads, and the samples were incubated at 65°C for 10 min. For total worm lysate, 15 adult worms were collected in 10 µl completed egg buffer as described above, 5 µl of Laemmli-buffer were added and the samples incubated at 65°C for 10 min. Lysates were run on 7.5% SDS gels and transferred onto nitrocellulose. GFP-tagged proteins were detected by incubating the membranes in anti-GFP-antibody (Torrey Pines Biolabs) in a 1∶5000 dilution in 3% BSA in TBS overnight at 4°C or for 2 hrs at RT. As secondary antibody, the HRP-coupled goat-anti rabbit serum from Thermo Scientific was applied in 1 1:15000 dilution in TBST for 1 hr. Signals were detected using ECL western blotting solution and ECL Hyperfilm (GE Healthcare).

### Fixation, Immunocytochemistry and Qdot®-staining

Immunofluorescence was done as described previously [16], with the following modifications: worms were rehydrated in PBS, 0.05% Tween-20 after methanol treatment and blocked for 30 min at RT in PBS, 2% BSA, 0.5% Tween-20 before incubation with the primary antibody. The SP-12::GFP worms were mounted in 5 µl of Citifluor AF1 (Citifluor Ltd.) directly after this treatment, since the GFP fluorescence was still well preserved. The tbb-2::GFP embryos were stained for 10 min with 1 µg/ml DAPI in PBS before being mounted. For the MAN-1::GFP embryos, an enhancement of the very weak GFP signal was achieved by incubating the slides for 2 hours at RT with mouse anti-GFP antibody (Roche Applied Science) 1∶100 in PBS, washing the slides 2 times for 5 min in PBS and subsequently incubating them with 1∶1000 AlexaFluor®488-coupled chicken anti-mouse secondary antibody (Life Technologies) for 45 min at RT in the dark. DAPI was added in this step at 1 µg/ml. After a final wash of 10 min in PBS, worms were mounted as described above and the slides sealed with nail polish. For the wheat germ agglutinin-Qdot®-labeling, the same fixation and blocking methods were used. Qdots® were applied at 5 nM concentration in PBS, 2% BSA over night at 4°C. The slides were washed for 20 min in PBS and mounted as described above.

### Live Sample Preparation and Staining

For pressure-free live embryo imaging, gravid 1-day adults were cut in a drop of egg buffer on a cover slip rimmed with vaseline, which was then placed upside-down on a 12-well diagnostic slide (‘hanging drop’ method) [17]. For whole-mount analysis, adult worms were placed on a 2% agar pad in a drop of 1 mM levamisole and covered with a cover slip. For the FM4-64 stained embryos, the *(ribo-1)RNAi* worms were cut as described above in 2 µg/ml FM4-64 in egg buffer, which was staining the permeable embryos instantly. The mock RNAi treated embryos were permeabilized by mounting the gravid adults on a dry 0.5% agar pad under light halocarbon oil, letting them dry for about 1 min and poking the embryos in the uterus gently with an injection needle. A 50-µl drop of FM4-64 was added, the worms were transferred to a 12-well slide using a mouth pipette and cut open to release the embryos. If successfully permeabilized, but not damaged, FM4-64 spread throughout the embryo in less than 1 min. For the developmental timing in Shelton’s Growth Medium, the medium was prepared as described by L.G. Edgar [18], using Cholesterol-3-Sulfate (Sigma) and 35% FCS.

### Imaging and Image Analysis

Epifluorescence images were acquired with a Zeiss Axioplan 2 microscope equipped with a Zeiss Axio Cam MRm camera (Carl Zeiss, Aalen Oberkochen, Germany) and a Plan Apochromat 63x/NA1.40 objective. Zeiss Axiovision 3.1 to 4.8 software was used to control hardware and process images. Confocal images were acquired on an Andor Revolution spinning disc confocal system (Andor, Belfast, UK) employing a Yokogawa CSU10 Scanner Unit and Andor iXon 885 CCD camera using an Olympus IX2-UCB inverted microscope with a 63× objective. Andor IQ2 software was used to control hardware and to acquire images. Post-processing and analysis was performed with ImageJ version 1.43u or Fiji version 1.47b. The Photomerge-function in Adobe Photoshop CS5 was used to assemble multi-image pictures of whole-mount worms.

### RNA Isolation, RT and Semi-quantitative PCR

Thirty adult worms fed for 48 hours on either mock or RNAi feeding plates were collected in a tube and washed two times with 1 ml egg buffer. The supernatant was aspirated, 1 ml Trizol (Invitrogen) was added and total RNA was isolated according to the manufacturer’s protocol. One vol. of 100% EtOH was added to the supernatant from the chloroform-extraction step. The mixture was loaded onto an RNeasy MinElute Cleanup column (Qiagen). The cleaning and elution was performed as described in the kit manual. 1 µg total RNA was treated with RQ1 RNAse-free DNAse (Promega) prior to cDNA first-strand synthesis employing oligo(dT)_15_-primer (Promega) and SuperScript II Reverse Transcriptase (Invitrogen) according to the manufacturer’s protocol. For the semi-quantitative PCR, the following intron-spanning primers were used:

5′-CACGATTCCCACTCTTTG-3′.

5′-TGGAGTGGCCACCTTTA-3′.

5′-TTCTCATTCTTGTCGTATTG-3′.

5′-GGAACCGATGGTATTGAAAG-3′.

5′-ATGCTCACCCTCACTCCAGC-3′.

5′-TCCGGACTCATCTCCATCG-3′.

5′-GCCAACACTGTTCTTTCCGG-3′.

5′-TCCAGACGGAGTACTTGCGC-3′.

PCR reactions were set up in a 25 µl volume containing 1 U Taq Polymerase (Roche), 0.25 µM of each primer and 0.12 µM dNTPs, using an equivalent of 50 ng total RNA for the OST-specific primers, respectively 5 ng for the actin-control. The reactions were run for 26 cycles in an MJ Mini Thermal Cycler (Biorad) with 55°C annealing temperature. Half of each reaction was loaded on a 3% agarose gel containing 1∶50,000 RedSafe nucleic acid stain (iNtRON Biotechnology). The DNA was visualized on a ChemiGenius^2^ gel imaging system (SynGene) operated by GeneSnap V6.07.

## Results

### The OST Complex is Essential for Development in *C. elegans*

**Figure 1:**
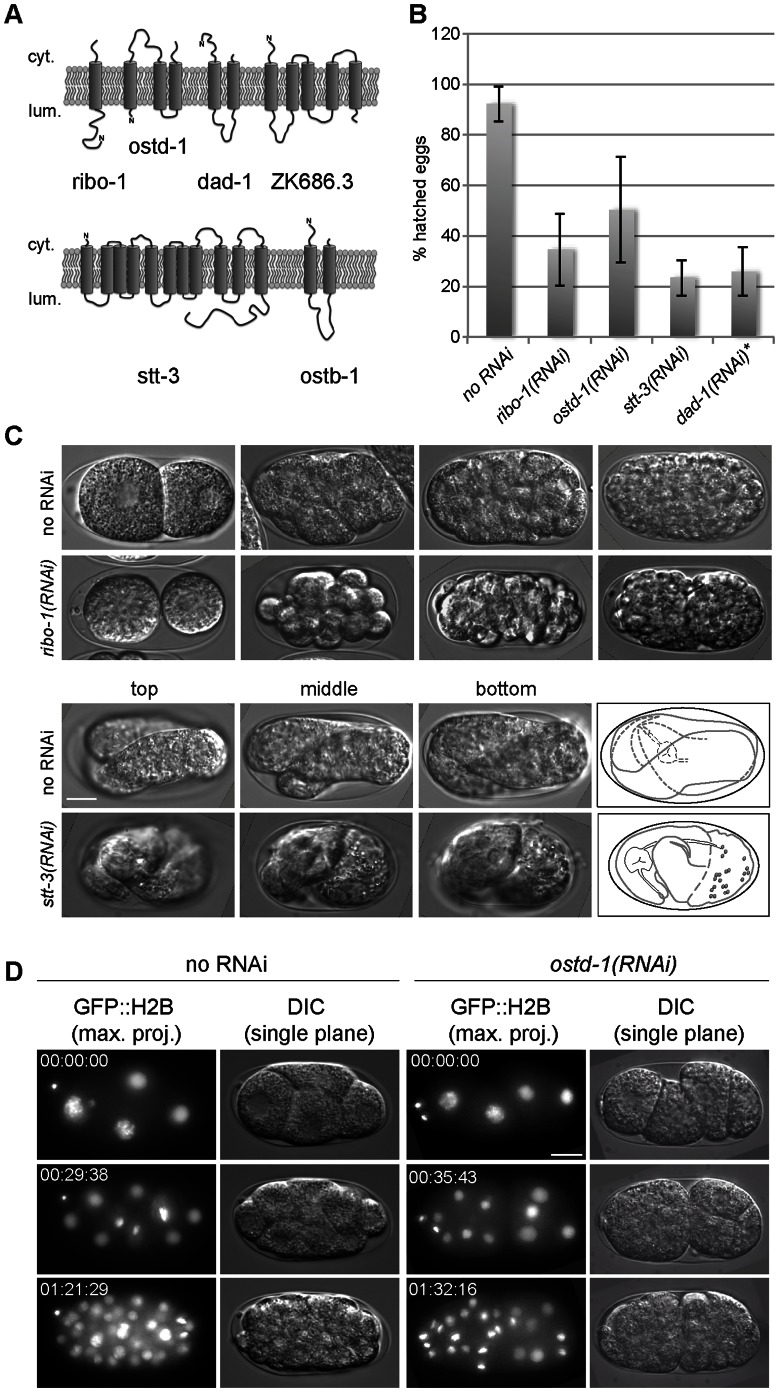
Knockdown of different OST complex members shows similar phenotypes. (A) Schematic representation of the membrane orientation of the *C. elegans* OST proteins in the ER membrane as calculated by *toppred 0.01*. (B) Embryonic lethality in OST complex member knockdown is similar in all four genes tested. Feeding was started at the L4 stage and carried on until worms stopped egg laying. *dad-1(RNAi*) was applied by dsRNA injection in young adult worms; as indicated by the asterisk. The ratio between total brood size and hatched larvae was determined. Error bars represent the standard deviation of at least 3 independent experiments. (C) Embryonic death did not occur at a specific stage in development upon knockdown of different OST complex subunits, but could happen any time before hatching. Early-arrested eggs showed characteristic rounded up cells as a consequence of the permeable eggshell and the slightly hyper-osmotic egg buffer. Late-arrested embryos could often twitch and showed distinct signs of morphogenesis like a pharynx, tail or gut, but also large vacuoles in the body as a sign of beginning necrosis. These phenotypes have been observed in more than 50% of the RNAied embryos in at least 3 independent experiments. (D) *OSTD-1* knockdown embryos developed slower than WT embryos as shown by 4-cell stage embryos that were left to develop on a slide in egg buffer at RT. Every 30 min a Z-stack image was taken. If the embryo did not arrest before, usually after 1.5 hours it showed approx. 30% less nuclei than the WT. Representative data from 4 independent experiments are shown. Scale bars represent 10 µm.

To understand the function of the OST complex in a developing multicellular organism, we knocked down the well-conserved components of the OST complex: ribophorin-1 (*C. elegans ribo-1*) and -2 (*ostd-1*), the catalytic subunit Stt3 (*stt-3*) and dad1 (*dad-1*) (Fig. 1A, Table 1). Similar to what has been reported previously [19], [20] in high throughput screens, knockdown of any of the four genes resulted in high embryonic lethality (Fig. 1B). The level of knockdown of different OST complex components is shown in Fig. S1. The embryos arrested in neither of the RNAi experiments at a particular time in development but continued cell division and differentiation until they appeared to ran out of critical factors that were needed for development (Fig. 1C). Still, the knockdown embryos developed slower than the mock control (Fig. 1D). Knockdown of OST complex components caused the embryos to be osmo-sensitive, and hence the cells rounded up in the egg-shell. To ensure that the developmental delay was not caused be the rounding up of the cells, we also employed blastomere medium, which should stabilize the cells. However, this medium also caused a similar developmental delay (Fig. S2A and B). Escapers were slow growing, clear, long, thin and uncoordinated. In agreement with this notion, increasing the knockdown efficiency, i.e. by starting feeding just after hatching (in the L1 stage of development), led to larval arrest at L3/L4. A minor fraction of the larvae reached adulthood but was sterile (data not shown). Since the phenotypes caused by knock-down of different subunits of the OST complex were virtually identical, we used for the analysis consistently *ribo-1(RNAi)*.

**Figure 2:**
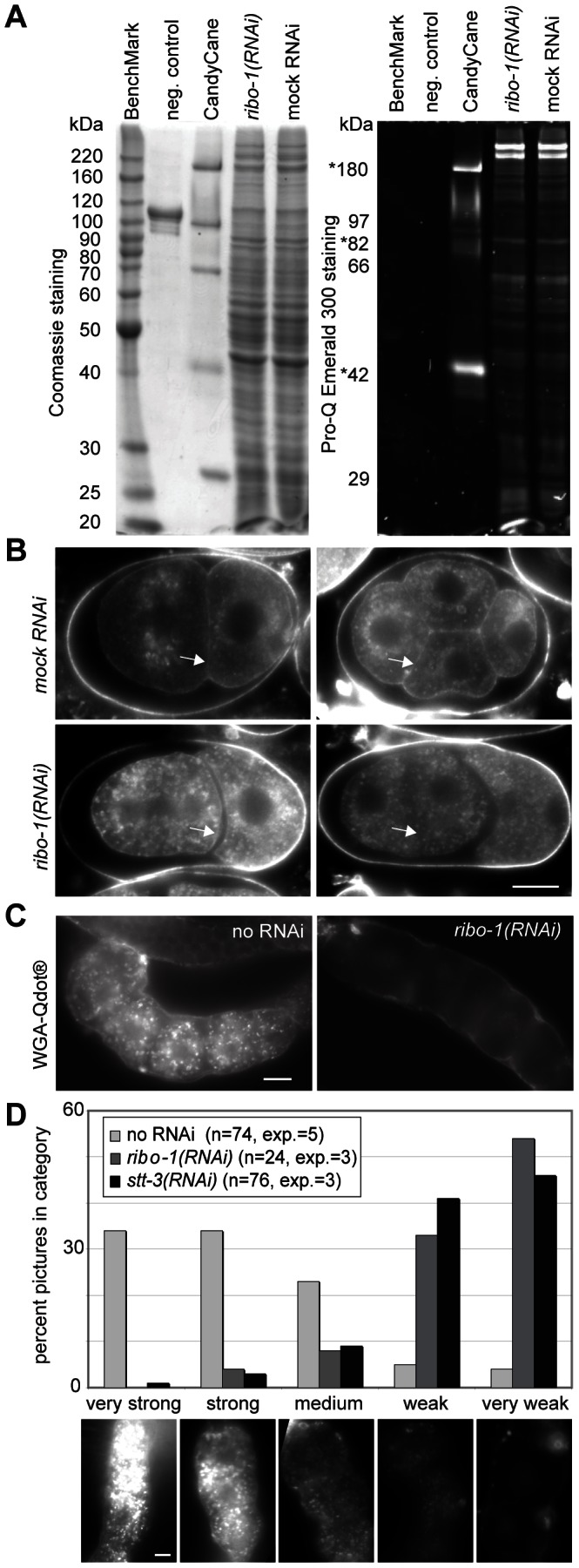
The amount of glycosylated proteins is reduced in oocytes but not embryos in *ribo-1(RNAi)*. (A) Downregulation of glycosylation cannot be detected in embryo lysates of *ribo-1(RNAi)*-treated worms. Both panels show the same gel loaded with 60 µg egg extract of either mock or *ribo-1(RNAi)*-treated embryos. Staining with ProQ Emerald 300 (Invitrogen) showed neither a reduction in staining nor an alteration of the band pattern in *ribo-1(RNAi)*, while subsequent Coomassie staining confirmed that equal amounts of protein were loaded. Asterisks indicate the glycosylated bands in the CandyCane marker. n = 3 independent experiments. (B) Epifluorescence images of fixed embryos stained with Qdot®-wheat germ agglutinin (Invitrogen) show a similar amount of cytoplasmic granules in mock vs. *ribo-1(RNAi),* but the plasma membrane staining (arrows) is absent in *ribo-1(RNAi)* embryos. n = 3 independent experiments (C) Identically fixed and stained WT oocytes contain brightly fluorescent granules in the cytoplasm which are fewer or absent in most of the *ribo-1(RNAi)* oocytes. (D) Quantification was performed by categorizing images according to the examples shown below. Scale bars represent 10 µm.

Knockdown of members of the OST complex should lead to a reduction of N-glycosylated proteins. To check this hypothesis, we isolated eggs from adult hermaphrodites that were fed with *ribo-1* dsRNA and analyzed the egg lysate for total and glycosylated protein content (Fig. 2A). Surprisingly, we could not detect any drastic reduction in the glycosylation pattern in *ribo-1(RNAi)* egg lysates. One possibility for this finding could be that the maternally contributed proteins were fully glycosylated, and they represent the major protein population in the total egg lysate. To investigate this possibility, we turned to an alternative approach and used the lectin wheat germ agglutinin (WGA) coupled to Q-dots as a fluorescent marker for N-glycosylated proteins. Again, the over all N-glycosylation levels appeared to be similar in early embryos. However, we noted that Q-dot staining was markedly reduced at the plasma membrane in *ribo-1(RNAi)* embryos (Fig. 2B, arrows). This result indicates that the knockdown might have worked at least partially in oocytes. Thus, we analyzed next the N-glycosylation levels in isolated gonads (Fig. 2C and D). Although the WGA Q-dots stained the sheath cells surrounding the gonad, the signal was strongly reduced in developing oocytes in *ribo-1(RNAi)* and *stt-3(RNAi)* compared to wild type, demonstrating that the knockdown was effective in oocytes. The biggest difference between an oocyte and an egg is that the sperm has fused with the oocyte to form a zygote. It has been shown previously that *C. elegans* sperm is resistant towards RNAi [17], [21]. Therefore, we propose that the sperm contains ER with functional OST complexes and that the paternally delivered OST complex partially rescues the early embryo and provides enough N-glycosylated proteins to drive cell proliferation at least for some time during development. This scenario would also provide an explanation why we did not observe a very strong arrest phenotype early on.

### The Knockdown of *ribo-1* Causes only Mild Effects on ER Morphology

**Figure 3:**
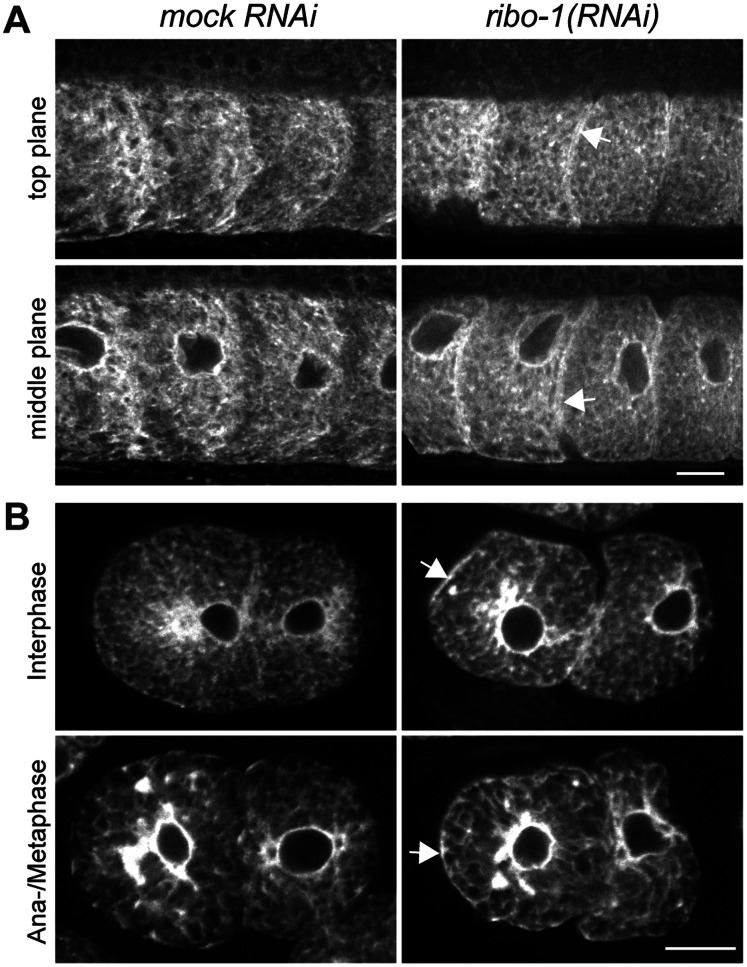
ER morphology is largely unaffected by N-glycosylation knockdown. (A) Spinning disc confocal microscopy of fixed whole mount GFP::SP12 worms showed that except for a slightly stronger accumulation at the cell-cell boundaries (arrows), the structure of the endoplasmic reticulum was not much altered in *(ribo-1)RNAi* oocytes, neither in the periphery (upper panels) nor in the center of the cells (lower panels). Images show the first four oocytes adjacent to the spermatheca, which would be to the left in all images. (B) Fixed GFP::SP12 embryos have been imaged spinning disc confocal microscopy. The only marked difference between mock and *(ribo-1)RNAi* was the stronger cortical accumulation (arrows), while the overall structure as well as the cycling of the ER between dispersed and sheet state was not affected. Scale bars represent 10 µm. n = ≥4 independent experiments.

Most of the secretory proteins are N-glycosylated, and N-glycosylation serves as a beacon for protein folding and proteostasis [22]. Therefore, the accumulation of un- or underglycosylated proteins could potentially result in morphological changes of the ER. To this end, we visualized the ER by expressing signal peptidase fused to GFP (SP12::GFP) [11]. The ER morphology in *mock(RNAi)* and *ribo-1(RNAi)* was comparable, with the only marked difference that we detected consistently more ER at the cortex of oocytes and early embryos upon *ribo-1(RNAi)*(Fig. 3). The ER cycles between a reticulate (interphase) and a sheet (meta- and anaphase) state in early *C. elegans* embryos [11]. These morphological changes were unaffected by *ribo-1(RNAi)*. Therefore, we conclude that reduced N-glycosylation only mildly affects ER morphology in *C. elegans* oocytes and early embryos.

### *ribo-1(RNAi)* Causes a Defect in Secretion

**Figure 4:**
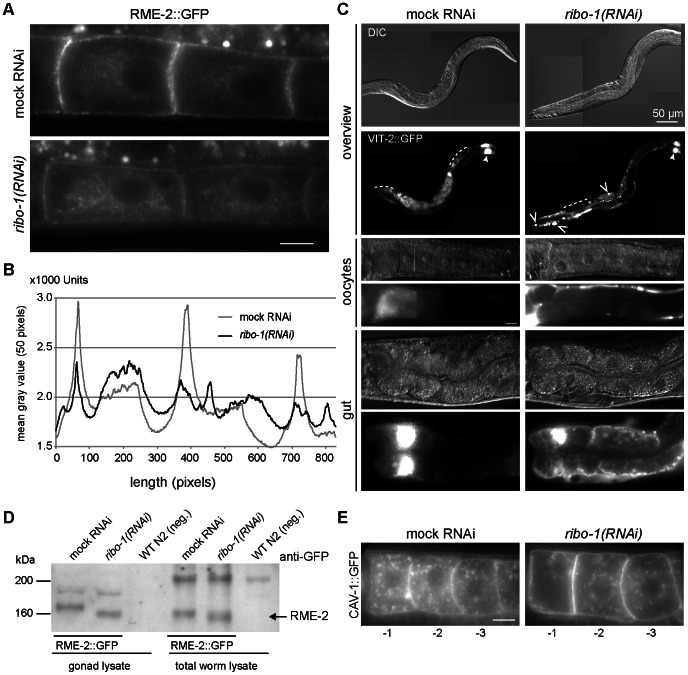
Secretion is impaired in *(ribo-1)RNAi*. (A) Epifluorescence images of live whole mount RME-2::GFP worms showed that the yolk receptor was no longer strongly accumulated at the plasma membrane of the oocytes in *(ribo-1)RNAi*. Depicted are the -2 and -3 oocytes adjacent to the spermatheca, which would be to the left in all images. (B) Quantification of the RME-2::GFP signal by ImageJ. A 50-pixel wide band was drawn over the center of the cells where the nuclei are located, and the mean gray value of every 50 pixel-column was plotted over the length of two cells. These line plots clearly illustrate the reduced plasma membrane accumulation of the yolk receptor in *ribo-1(RNAi)* oocytes. (C) VIT-2::GFP accumulated in the body cavity of *(ribo-1)RNAi* worms (open arrow heads and middle panels) while the oocytes were practically devoid of VIT-2::GFP staining (dashed lines and middle panels). Moreover, the lowest panels show that yolk protein was not even efficiently secreted from the gut cells, as they appeared much brighter in the *(ribo-1)RNAi* worms, pointing towards a general defect in secretion. These phenotypes have been observed in more than 80% of the RNAied worms, in 3 independent experiments. The two very bright gut cells right next to the pharynx (filled arrow heads in upper panel) can be found in many GFP worm lines. (D) Immunoblots of worm lysates developed with an anti-GFP antibody detect an increased electrophoretic mobility of RME-2::GFP protein upon *ribo-1(RNAi)*. This phenotype was present in lysates from isolated gonads as well as in total worm lysate from RME-2::GFP-tagged worms, while the absence of a similar band in the N2-lysate demonstrates its specificity. Also, RME-2::GFP has a calculated mass of approximately 130 kDa, while the bands we detected run at around 160 kDa, indicating that RME::2 is probably modified. Upon OST knockdown, these modifications are altered, leading to a different electrophoretic mobility. (E) CAV-1::GFP secretion is not impaired in *ribo-1(RNAi)* worms. Live imaging CAV-1::GFP tagged worms demonstrate that there is not a general block in secretion upon knockdown of the OST complex. The CAV-1::GFP staining in mock treated and *ribo-1(RNAi)* oocytes is comparable. Scale bars in all panels represent 10 µm, if not annotated differently.

Given the mild the phenotype of *ribo-1(RNAi)* on ER morphology, we wanted to test next, whether the reduction in N-glycosylation would impair secretion. The first hint that this might indeed be the case, came form the observation that the embryos were osmo-sensitive (Fig. 1C) causing rounding up of the cells in the egg-shell. In oocytes, the yolk receptor RME-2 is exported to the plasma membrane, endocytosed upon binding of yolk protein, and recycled back to the surface through recycling endosomes [10]. Yolk is produced in the gut epithelium and secreted into the pseudocoelomic space. To monitor yolk secretion from the gut epithelium and uptake into oocytes, we use the yolk protein VIT-2 fused to GFP as a marker [10]. At steady state RME-2::GFP is concentrated at the plasma membrane of oocytes, with a large intracellular pool [10], [23] (Fig. 4). This plasma membrane localized pool of RME-2::GFP was reduced upon *ribo-1(RNAi)*, consistent with a defect in secretion (Fig. 4A and B). Consequently, VIT-2::GFP uptake was impaired as it accumulated in the body cavity (Fig. 4C). Moreover, a VIT-2::GFP secretion defect was also observed in the intestine, leading to the accumulation of yolk protein in epithelial cells (Fig. 4C). The yolk receptor RME-2 is glycosylated. We therefore tested, whether we could detect reduced glycosylation of RME-2 in *ribo-1(RNAi)* gonads. The electrophoretic mobility of RME-2::GFP was probed in lysates of gonads or entire worms either mock or *ribo-1(RNAi)* treated by immunoblot (Fig. 4D). RME-2::GFP migrated faster in the SDS PAGE in lysates from *ribo-1(RNAi)* treated animals, consistent with a lower molecular weight and the lack of N-glycosylation. In contrast the plasma membrane localization of the non N-glycosylated protein caveolin CAV-1 was unaffected by knockdown of RIBO-1 (Fig. 4E). Taken together, these data support the notion that proper N-glycosylation is required for secretion in different tissues in *C. elegans*.

### RIBO-1 Knockdown Causes Severe Cytokinesis Defects

**Figure 5:**
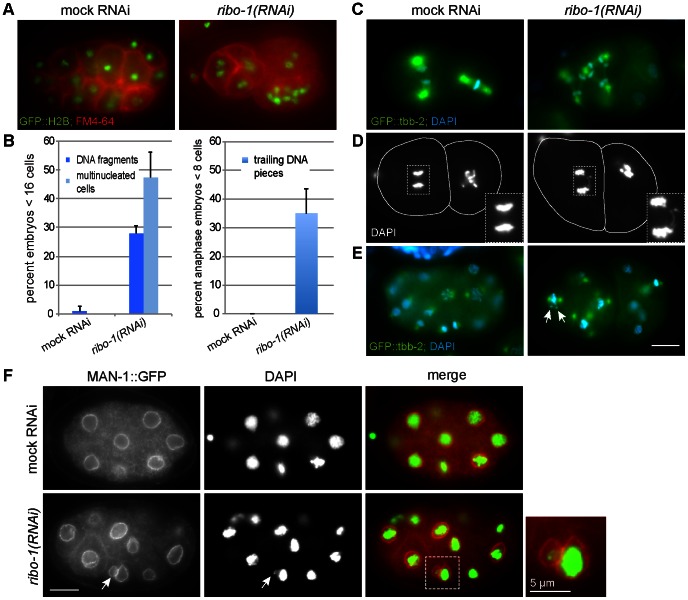
N-Glycosylation knockdown causes severe defects in chromosome segregation and cytokinesis. (A) Live permeabilized H2B::GFP embryos stained with FM4-64 showed an increased number of nuclei accumulating in cells of the *(ribo-1)RNAi* embryos. (B) Quantification of the phenotypes shown in A, C, D and E. n = >3 independent experiments with >30 embryos/experiment were counted. Only embryos earlier than 16-cell stage were analyzed for the cytokinesis defect. For the analysis of trailing DNA pieces only cells in ana- or telophase of embryos in the 1–8 cell stage were taken into account (n = >3 independent experiments), and a DNA filament had to be visible between the two DNA masses to be classified positive in the sense of the phenotype. As DNA fragments we classified small DAPI- or GFP-stained spots in the cytoplasm at a marked distance away from the nuclei, as shown in (E) and (F) (arrows). As multiple nuclei we counted cells that showed accumulations of two or more DNA masses of similar size next to each other. (C) Fixed tbb-2::GFP embryos stained with DAPI. The accumulated nuclei in the anterior cell of the *(ribo-1)RNAi* embryo divided simultaneously, the spindle microtubules interconnecting several centrosomes to form an extended spindle network throughout the cell. (D) Fixed 2-cell stage embryos stained with DAPI, showing the AB cell in anaphase. In the *(ribo-1)RNAi* embryo, the clearly visible DNA thread connecting the two DNA masses indicated that a chromosome had been attached to microtubules from both spindle poles during Metaphase and now has been pulled apart. (E) Fixed tbb-1::GFP embryos stained with DAPI. Arrows point to two DNA fragments next to a metaphase nucleus, which seem not to be arranged on the metaphase plate. These might be the remnants of a previous mis-segregation event. (F) MAN-1::GFP embryos stained with anti-GFP and DAPI. Arrows point to a small piece of DNA that attracted nuclear envelope components and thus formed a micronucleus. The content of the dashed-line box was magnified. Scale bars, if not differently annotated, represent 10 µm.

Cytokinesis requires the deposition of new plasma membrane as the cleavage furrow ingresses during cell divisions. Given that we observed a secretion defect in *ribo-1(RNAi)* oocytes we asked next, whether reduction of properly N-glycosylated proteins at the plasma membrane (Fig. 2B) would affect cytokinesis in early embryos. Interestingly, most *ribo-1(RNAi)* one-cell stage embryos were able to divide into two cells. However, subsequent cytokinesis events failed more often. About 50% of the embryos showed at least one if not multiple cytokinesis failures as indicated by staining the plasma membrane with the lipophilic dye FM4-64 and the DNA with GFP::H2B (Fig. 5A). Similar results were obtained using the plasma membrane marker PH(PLC)::GFP and DAPI staining (data not shown). As a result, those embryos contained multinucleated cells, and the centrosomes in those cells formed extensive interconnecting microtubule networks (Fig. 5C) in 26.9% ±5.41% of embryos.

### RIBO-1 and OSTD-1 Knockdowns Cause Chromosome Missegration and DNA Trailing

We noticed that in some of the cells in *ribo-1(RNAi)* embryos pieces of DNA appeared to be retarded in the segregation during anaphase (Fig. 5A). We first checked in two-cell stage *ribo-1(RNAi)* embryos, whether we could observe defects in DNA condensation and/or chromosome segregation by staining DNA with DAPI (Fig. 5D). During anaphase of the AB cell, we observed DNA fragments that were trailing behind after the meta-anaphase transition. As a consequence, pieces of DNA were observed in embryos that were not aligned on the mitotic spindle in ribo-1 (Fig. 5B and E, arrows) and were left outside of the newly forming nucleus in telophase (Fig. 5F). A similar effect was observed when we knocked down OSTD-1 (Fig. S2B). Our data indicate a novel and unexpected role for N-glycosylation in chromosome segregation, independent of its function in cytokinesis.

## Discussion

We have analyzed the effects of reduced N-glycosylation on early development of *C. elegans* and found that like in yeast, N-glycosylation is an essential process. N-glycosylation was already required for proper oogenesis, however employing a less stringent RNAi protocol allowed us to follow the effect of reduced N-glycosylation on development. Under these conditions, the embryos did not arrest at a particular time point i.e. at the one- or two-cell stage, but rather continued throughout development until they ran out of crucial factors. This pleiotropic arrest can be expected considering the large variety of different protein clients for the OST complex. The relatively late state of arrest also implies that the OST complex per se is probably relatively stable and that the paternally delivered OST complex is sufficient to N-glycosylate proteins for a relatively long time in development. This finding would also suggest that for at least some proteins, it would suffice to perform their cellular function if only a sub-fraction was properly N-glycosylated. However, it should be noted, that despite the relatively late arrest of the *OST(RNAi)* embryos, multiple problems were already detected before the arrest, most notably defects in cytokinesis, preventing the accurate development of the embryo.

The cytokinesis defect is most likely a consequence of the reduction in intracellular traffic and secretion. Secretion is generally affected because 1. the yolk receptor RME-2 did not reach efficiently the oocyte plasma membrane, 2. the egg shell was not correctly formed after fertilization yielding osmo-sensitive embryos due to a reduction in secretion of egg shell material. 3. yolk protein, which is synthesized in the intestine was only partially secreted as some of it was retained in the gut epithelial cells.

We also observed significant yolk protein accumulating in the pseudocoelomic space, which could not be taken up by RME-2 in the oocytes. There are two explanations for the phenotype. Either very little RME-2 reached the plasma membrane and also its internal cycling might have been slowed down, or the lack of N-glycosylation prevented the efficient interaction with yolk. We cannot distinguish between these two possibilities. The matter is even more complicated by the fact that yolk protein itself is a glycoprotein [24].

The reduction of N-glycosylation should induce ERAD and un- or misfolded proteins would accumulate in the ER. This scenario might lead to a change in ER morphology, similarly to a block of secretion. However, we only detected a minor defect in *ribo-1(RNAi)* embryos and oocytes, and the cycling between the sheet and the reticulate state of the ER during the cell cycle was normal. It is conceivable that we cannot detect gross defects because there is no massive accumulation of un- or misfolded proteins. In developing oocytes, the secretory pathway is probably not very active and hence ERAD might be easily able to deal with the protein load in the ER, and in the early embryos, we may not observe much due to the paternal contribution of the OST complex. In addition some cargo may leave the ER despite the lack of N-glycosylation. The ER cannot control for functionality. Therefore, a subset of proteins may leave the ER because they adopted a fold, which buried most of the hydrophobic residues, and hence may appear to be correctly folded.

Most surprisingly, chromosome segregation was disturbed in a large number of embryos as we detected small DNA masses outside nuclei. This observation could be due either to failure to attach all chromosomes onto microtubules in metaphase or to a segregation defect at the metaphase-anaphase transition. The latter possibility appears to take place because we can detect trailing chromosomes. We cannot exclude a defect in chromosome attachment. However, the kinetochores in *C. elegans* are holocentric [25], [26] and hence multiple microtubule attachment sites are present on an individual chromatid. Why would a reduction in N-glycosylation result in a chromosome segregation defect? Golgi, endosomes and lysosomes are gathered around centrosomes at the spindle poles because of their interaction with (-) directed motor proteins. However, it seems unlikely that they would interfere with chromosome segregation. In contrast, ER membranes cover the mitotic spindle, which is most prominent in anaphase [11]. The function of this attachment is unclear, but it can be speculated that these ER membranes also contain nuclear pore complex components. At least the MAN-1, a nuclear envelope marker [13] is localized at this stage in a manner indistinguishable from the ER markers HDEL-GFP or SP-12::GFP [11], [27] (Fig. 5). These membranes wrap around chromosomes already during the movement towards the poles [11]. In Drosophila syncytial embryos, ER can also be found on spindles and in this case generate Ca^2+^ microdomains; these Ca^2+^ transients appear to be necessary for nuclear division [28]. Therefore, one explanation could be that upon *ribo-1(RNAi)* we would interfere with Ca^2+^ signaling at the ER. But there are many other possibilities as the effect that we observe could also be very indirect. For example, in cancer cells it has been shown that unliganded progesterone receptors, which are predicted to be N-glycosylated, were still signaling competent and could modulate the spindle assembly checkpoint through changes in gene expression [29]. It will be important in the future to determine how chromosome segregation is dependent on N-glycosylation.

## Supporting Information

Figure S1 **OST complex members can be knocked down efficiently by feeding RNAi.** Semi-quantitative PCRs using intron-spanning primers were performed on cDNA derived from 30 adult worms that were either RNAi or mock treated by feeding for 48 hours. Equal volumes of each PCR were run on a 3% agarose gel, showing clearly an almost complete knockout of *ribo-1* and *stt-3*, as well as an approximately 50% knockdown of *ostd-1*.
(TIF)

Figure S2 ***ostd-1(RNAi)*** **causes developmental delay in early embryos.** (A) *OSTD-1* knockdown embryos developed slower than mock-treated embryos as shown by 4-cell stage embryos tagged with H2B::GFP that were left to develop on a slide in Shelton’s Growth Medium (SGM) at RT. Every 30 min a Z-stack image was taken and the nuclei were counted. We assembled the values of each 7 different embryos over four time points, demonstrating that despite the isosmotic buffer conditions, the RNAi-treated embryos still developed slower than their mock-treated counterparts. Error bars depict the standard deviation, and the large deviation in the case of the *ostd-1(RNAi)* comes from the fact that about half of the embryos were arrested after 60 minutes. (B) SGM did neither rescue the chromosome segregation defects nor the cytokinesis failures, as shown by examples from an *ostd-1(RNAi)* time course, where at the 60 minutes – time point we observed an anaphase with trailing DNA pieces (arrow), which later in the 90 minute – time point resulted in a cell containing two nuclei and two micronuclei (arrowheads). The scale bar represents 10 µm.
(TIF)
