# Supplementary figures and images for: N-Glycosylation Is Required for Secretion and Mitosis in C. elegans
Source: PLoS One. 2013 May 14;8(5):e63687. doi: 10.1371/journal.pone.0063687 (PMC3653792; doi:10.1371/journal.pone.0063687)

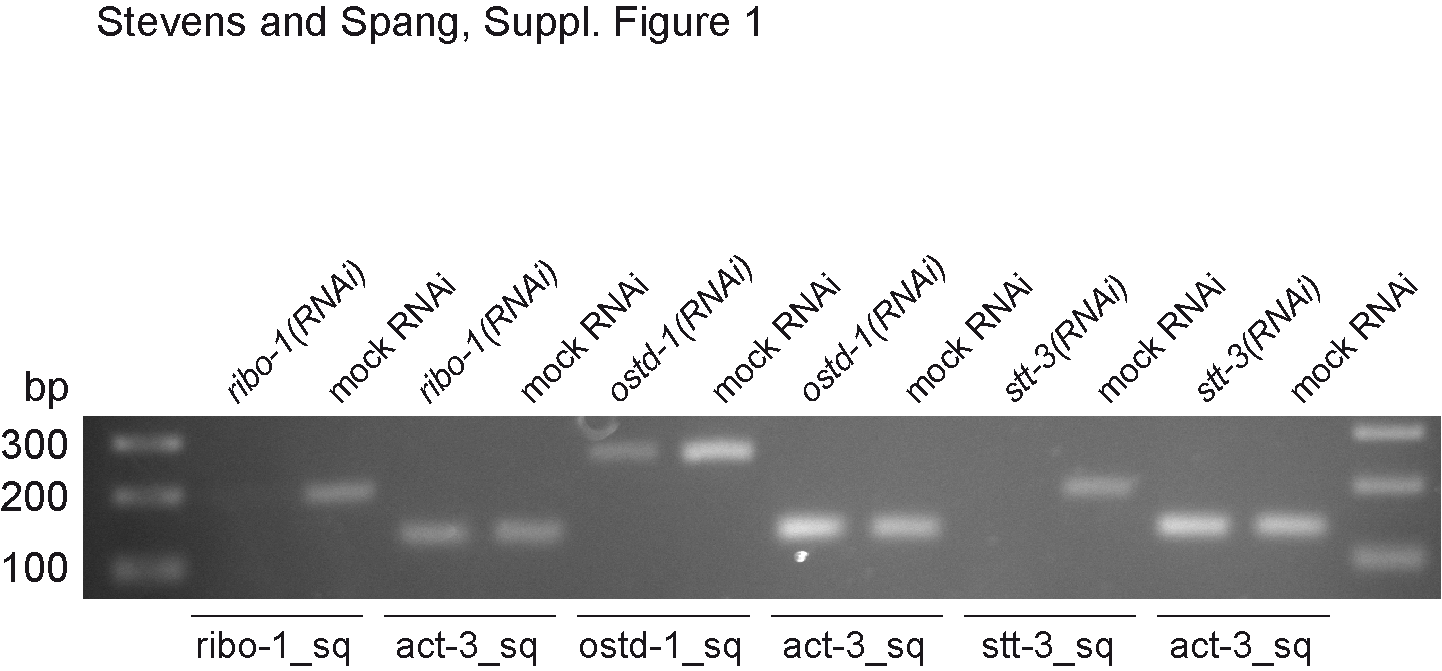

Supplement: Figure S1 — OST complex members can be knocked down efficiently by feeding RNAi. Semi-quantitative PCRs using intron-spanning primers were performed on cDNA derived from 30 adult worms that were either RNAi or mock treated by feeding for 48 hours. Equal volumes of each PCR were run on a 3% agarose gel, showing clearly an almost complete knockout of ribo-1 and stt-3, as well as an approximately 50% knockdown of ostd-1. (TIF) [file pone.0063687.s001.tif]

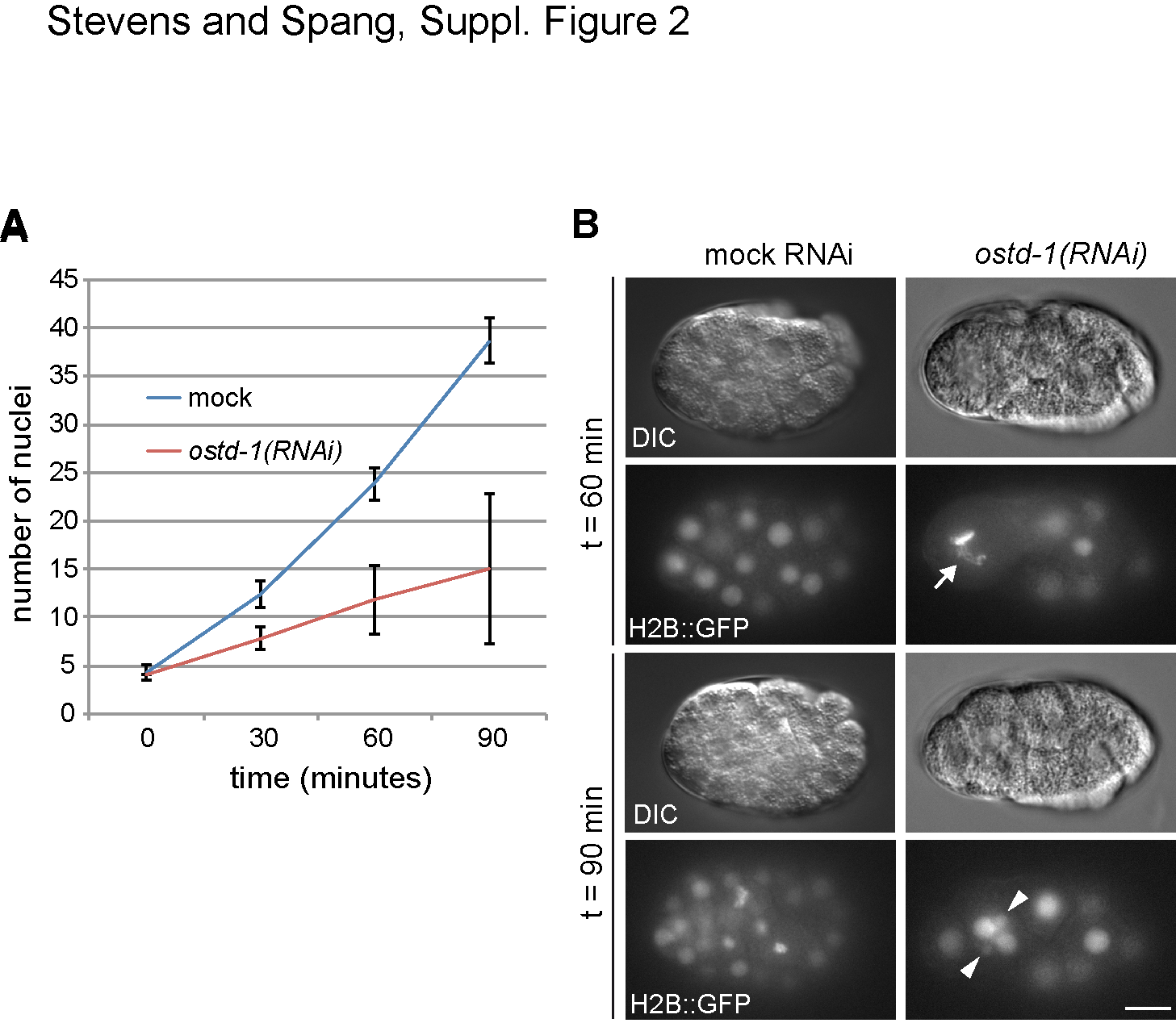

Supplement: Figure S2 — ostd-1(RNAi) causes developmental delay in early embryos. (A) OSTD-1 knockdown embryos developed slower than mock-treated embryos as shown by 4-cell stage embryos tagged with H2B::GFP that were left to develop on a slide in Shelton’s Growth Medium (SGM) at RT. Every 30 min a Z-stack image was taken and the nuclei were counted. We assembled the values of each 7 different embryos over four time points, demonstrating that despite the isosmotic buffer conditions, the RNAi-treated embryos still developed slower than their mock-treated counterparts. Error bars depict the standard deviation, and the large deviation in the case of the ostd-1(RNAi) comes from the fact that about half of the embryos were arrested after 60 minutes. (B) SGM did neither rescue the chromosome segregation defects nor the cytokinesis failures, as shown by examples from an ostd-1(RNAi) time course, where at the 60 minutes – time point we observed an anaphase with trailing DNA pieces (arrow), which later in the 90 minute – time point resulted in a cell containing two nuclei and two micronuclei (arrowheads). The scale bar represents 10 µm. (TIF) [file pone.0063687.s002.tif]
